# ^1^H-MRS study of hippocampus in advanced prostate cancer patients: Relationship between hippocampal secondary damage and cognitive disorder following combined androgen blockade therapy

**DOI:** 10.1371/journal.pone.0323323

**Published:** 2025-05-07

**Authors:** Peng Guo, Xiaoming Xing, Keli Wu, Yu Wang, Zhibin Chen, Liang Cao, Xiaorong Li, Ning Li

**Affiliations:** 1 Department of Urology, Neijiang First People’s Hospital, Neijiang, Sichuan, China; 2 Department of Radiology, Neijiang First People’s Hospital, Neijiang, Sichuan, China; 3 Department of Neurology, Neijiang First People’s Hospital, Neijiang, Sichuan, China; 4 Department of Pathology, Neijiang First People’s Hospital, Neijiang, Sichuan, China; University of Modena and Reggio Emilia, ITALY

## Abstract

**Objectives:**

To determine whether 6 months of combined androgen blockade (CAB) treatment would result in metabolic changes of hippocampus and whether metabolic changes correlate with changes in cognition in patients with advanced prostate cancer (PCa).

**Materials and methods:**

This is an observational study. Proton magnetic resonance spectroscopy (1H-MRS) was used to observe the changes in the ratios of N-acetylaspartate/creatine (NAA/Cr) and choline-containing compounds/creatine (Cho/Cr) on the bilateral hippocampus for the patients before and 6 months after CAB treatment. Cognitive function was also assessed by the Beijing version of the Montreal Cognitive Assessment (MoCA-BJ) at the above two time points. Additionally, a certain number of matched individuals undergoing physical examination were selected as the control group.

**Results:**

CAB group comprised 25 patients with advanced PCa completing follow-up, while control group had 22 healthy controls. Prior to CAB, no significant differences existed in MoCA-BJ scores (including sub-scores) or bilateral hippocampal NAA/Cr and Cho/Cr ratios between groups. Six months after CAB, CAB group exhibited marked declines in MoCA-BJ total score, delayed recall, visuospatial/executive, and attention functions, alongside reduced bilateral hippocampal NAA/Cr and elevated left hippocampal Cho/Cr (*P* < 0.05).The results of multiple linear regression indicate a positive correlation between NAA/Cr in the left hippocampus and MoCA-BJ total score (*β* = 4.66, *P* < 0.001), as well as delayed recall function (*β* = 2.76, *P* < 0.001). Mediation analysis confirms that testosterone influences the MoCA-BJ total score and delayed recall function by affecting NAA/Cr in the left hippocampus.

**Conclusions:**

The impact of advanced PCa on cognitive performance could be negligible. However, patients experienced secondary hippocampal injury after CAB, which further led to cognitive dysfunction.

## Introduction

It has been proved that the hippocampus is a central nodal brain region regulating multiple cognitive domains, especially playing a crucial role in human learning and memory [1]. Androgens play a vital role in maintaining normal biochemical metabolism of hippocampal neurons, inducing the growth of hippocampal dendritic spines, and the formation of synapses [2]. The mechanisms underlying this are thought to be related to the following factors. First, serum testosterone levels are positively correlated with blood flow in the corresponding brain regions of the hippocampus. A decrease in serum testosterone levels can lead to a reduction in blood flow to the hippocampus [3], resulting in apoptosis of hippocampal neurons [4–6]. Second, androgen receptors (ARs) are widely distributed in the hippocampus [7]. The expression of these ARs is not limited to the nucleus, but also exists outside the nucleus, such as cell membrane, mitochondria and synaptic vesicles [8].Androgen is now known to act via genomic and nongenomic receptors. In many cases, the same receptor molecule has different functions in the nucleus and non-nuclear sites in the cell [9]. Moreover, literature has reported that androgens, including testosterone and its metabolite dihydrotestosterone (DHT), have neuroprotective effects, which can improve Alzheimer’s disease (AD)-related neuropathology and cognitive performance [10]. Their neuroprotective effects are exerted through the following mechanisms: anti-apoptosis, anti-oxidative stress, and anti-neuroinflammatory effects [10–12]. It has been observed that normal aging was associated with significant decreases in androgens in male brains [13]. The literature also reports that testosterone levels in male patients with AD and male patients with depression are significantly lower than those in normal males [13,14].

Prostate cancer (PCa) is one of the most common malignant tumors in men. PCa initiation and progression is driven by androgens through binding to the AR. The incidence of PCa is also increasing year by year in China [15]. However, due to various factors, most patients with PCa in China are already in the advanced stage when they seek medical treatment, losing the indication for radical surgery, and can only choose endocrine therapy. Research shows that the levels of systemic inflammatory serum markers such as Interleukin-6 (IL-6) and C-reactive protein (CRP, whose production is stimulated by IL-6) in patients with advanced PCa are significantly higher than those in patients with early PCa and normal elderly men [16–18].The question of whether elevated levels of IL-6 and CRP in serum definitively precipitate neuroinflammatory responses, neuronal deterioration, and cognitive impairments remains inconclusive, with research outcomes presenting a contradictory landscape [19–23].Current guidelines recommend that for advanced PCa patients, androgen deprivation therapy (ADT) combined with docetaxel or new androgen receptor axis-targeting (ARAT) agents like abiraterone, enzalutamide, darolutamide or apalutamide should be the first-line treatment [24,25]. However, due to the exorbitant prices of new ARAT drugs and the deficiencies in the medical insurance system, many patients in China, especially in the vast central and western regions, still opt for traditional combined androgen blockade (CAB) therapy, the addition of bicalutamide to castration (surgical or medical), as their initial treatment plan.CAB was found to improve both progression-free survival (PFS) and overall survival (OS), compared with ADT alone in advanced PCa patients [26].

However, it has not been reported yet whether the sharp decline in androgen levels and the blockade of ARs may cause damage to the biochemical metabolism of the hippocampus in patients, subsequently leading to cognitive impairment (CI). Proton magnetic resonance spectroscopy (^1^H-MRS) is a non-invasive diagnostic technique that enables the detection, localization, and quantitative analysis of dynamic metabolic changes in living brain tissue. Currently, this technology has been widely utilized to investigate metabolic changes in the brain tissue of patients with various conditions, including AD, Parkinson’s disease, diabetes, depression, and mania [27]. The ratios of N-acetylaspartate/creatine (NAA/Cr) and choline-containing compounds/creatine (Cho/Cr), with Cr as a reference, can effectively reflect the changes in the concentrations of NAA and Cho. Literature reports have shown that patients with mild cognitive impairment (MCI), AD, and cognitive dysfunction due to stroke have significantly decreased NAA/Cr ratios in the bilateral hippocampi compared with normal individuals, while Cho/Cr ratios are elevated [28–30].To date, there have been no reported studies utilizing ^1^H-MRS to investigate the alterations in hippocampal metabolism among advanced PCa patients who have undergone long-term CAB therapy. Therefore, we designed this observational study with two primary objectives: First, to compare the cognitive function and hippocampal metabolism between patients with advanced PCa and normal elderly males. Second, to compare the cognitive function and hippocampal metabolism of advanced PCa patients before and after six months of CAB therapy.

## Participants and methods

This is an observational study that began on April 1st, 2022. The selection criteria for all participants are as follows:

### Inclusion criteria

#### Prostate cancer group.

(1) From April 1st, 2022 to December 31st, 2023, male patients aged 60 and above, who are right-handed, were hospitalized in the Urology Department of the First People’s Hospital of Neijiang(Neijiang, China); (2) Diagnosed with PCa through prostate biopsy under local anesthesia, with locally advanced or metastatic PCa indicated by chest and abdominal CT, pelvic MRI, and whole-body bone scan; (3) The initial treatment plan is suitable for CAB (leuprolide acetate injection 3.75mg, subcutaneous injection, once every 28 days; combined with oral bicalutamide capsule 50mg, once daily); (4) Sufficient vision and hearing to undergo neuropsychological testing; (5) The patient agrees to participate in this study.

#### Control group.

(1) From April 1st, 2022 to December 31st, 2023, males who underwent physical examination at Neijiang First People’s Hospital’s physical examination center have normal serum levels of prostate specific antigen (PSA); (2) Aged 60 years or above, right-handed;(3) Possessing visual and auditory abilities sufficient for neuropsychological testing; (4) The research participant agrees to participate in this study.

### Exclusion criteria

(1) Subjects with various neurological diseases that can affect cognitive function, including AD, vascular dementia, stroke, Parkinson’s disease, inflammatory diseases of the central nervous system, intracranial tumors, epilepsy, etc.;(2) Subjects with immune system diseases, such as hyperthyroidism, hypothyroidism, systemic lupus erythematosus, etc.;(3) Subjects with claustrophobia or who have metal implants such as pacemakers that prevent them from undergoing MRI examinations;(4) Subjects with a history of psychiatric disorders such as depression or mania, or a family history of mental illness;(5) Subjects with a history of alcoholism, drug abuse, or long-term use of glucocorticoids, antipsychotic drugs, sedatives, or hypnotics that can affect cognitive function;(6) Patients with PCa that has progressed to the castration-resistant prostate cancer (CRPC) stage and switched to other anti-androgen drugs;(7) Diabetic subjects;(8) Illiterate subjects;(9) Subjects who have received prior systemic chemotherapy or intracranial radiotherapy.

### Research methodology

1) Data was collected from the research participants, including their age, years of education, history of hypertension, smoking history, Body Mass Index (BMI), TNM staging for PCa patients, initial blood testosterone levels for all participants, blood testosterone levels for PCa patients six months after CAB treatment, initial serum Prostate Specific Antigen (PSA) levels for all participants, serum PSA levels for PCa patients six months after CAB treatment, initial serum Hypersensitive C-reactive Protein (hs-CRP) levels for all participants, and serum hs-CRP levels for PCa patients six months after CAB treatment. The follow-up intervals and examination items for PCa patients after CAB treatment were conducted according to the recommendations of the 2021 European Association of Urology (EAU) Guidelines [25].
2) The cognitive function of these participants was evaluated by a standardized language in one-on-one interviews conducted by the same professionally trained neurologist using the Beijing version(www.mocatest.org) of the Montreal Cognitive Assessment (MoCA-BJ). After conducting a large-scale survey, Lu and his team concluded that the optimal cutoff points were 14 for illiterate individuals, 20 for individuals with 1–6 years of education, and 25 for individuals with 7 or more years of education in Chinese elderly individuals, and 1 point is added to the test score to correct for the influence of age if the subject is ≥ 90 years [31]. During the assessment, efforts were made to minimize interference from the surrounding environment to ensure the reliability of the scale’s evaluation results. Patients in the PCa group underwent two assessments: the first upon enrollment into the group, and the second six months after they began receiving CAB therapy. In contrast, participants in the control group underwent only one assessment.
3) Using the Siemens MAGNETOM Skyra 3.0T superconducting magnetic resonance imaging system equipped with an eight-channel scanning coil, a head scan was performed to receive and acquire magnetic resonance data. Initially, conventional T1WI, T2WI, and T2-FLAIR sequences were carried out to screen for any other abnormal lesions in the head. Following this, bilateral hippocampal ^1^H-MRS imaging was performed. On the axial plane, the center of the bilateral hippocampal body was identified as the region of interest (ROI). The ^1^H-MRS data was acquired using a three-dimensional multi-voxel acquisition method with the following parameters: TR 1700 ms, TE 135 ms, flip angle 90°, slice thickness 1.5 mm, FOV 120 mm × 120 mm, voxel size 10 mm × 10 mm × 15 mm. The scan time was 223 seconds. The voxel volume setting was generally determined based on individual differences, and the bilateral ROIs for the same individual should be of the same size. ^1^H-MRS Data were collected using the software provided by Siemens. After data collection was completed, the acquired data were further processed and analyzed using the Syngo.via post-processing software. The area under the curve of each metabolite peak was calculated, and creatine (Cr) was used as an internal reference to calculate the relative concentrations of N-acetylaspartate/creatine (NAA/Cr) and choline-containing compounds/creatine (Cho/Cr). To avoid errors, the same area was measured three times repeatedly, and the arithmetic mean was taken as the final measurement value. Patients in the PCa group underwent two head MRI scans: the first before CAB treatment and the second six months after CAB treatment. Participants in the control group underwent only one head MRI scan.

This study was approved by the Ethics Committee of Neijiang First People’s Hospital (Neijiang, China; approval number: 2021-LSP-53) and registered at the Chinese Clinical Trial Registry (No.: ChiCTR2200056495). All participants have signed a written informed consent form and were free to withdraw from the study at any time during the research period.

### Statistical analyses

Analysis was performed using R 4.2.1 (http://www.Rproject.org; The R Foundation, Vienna, Austria) and the Free Statistics software (version 2.0; Beijing Free Clinical Medical Technology Co., Ltd, Beijing, China) [32]. Quantitative data between the two groups were analyzed using an Student’s *t*-test if they conformed to a normal distribution and had homogeneity of variance; otherwise, a rank sum test was used. Paired sample t-tests were used to compare quantitative data from the PCa group before CAB and 6 months after CAB therapy. Qualitative data were converted into a 2 × 2 contingency table and analyzed using the chi-square (χ²) test. The Spearman correlation analysis was used to analyze the correlation between two variables. For positive results obtained from the Spearman correlation analysis, if they met the conditions for multiple linear regression, a multiple linear regression analysis was conducted to confirm the presence of a true correlation, while excluding the interference of covariates. A *P*-value of less than 0.05 was considered statistically significant.

## Results

**Fig 1:**
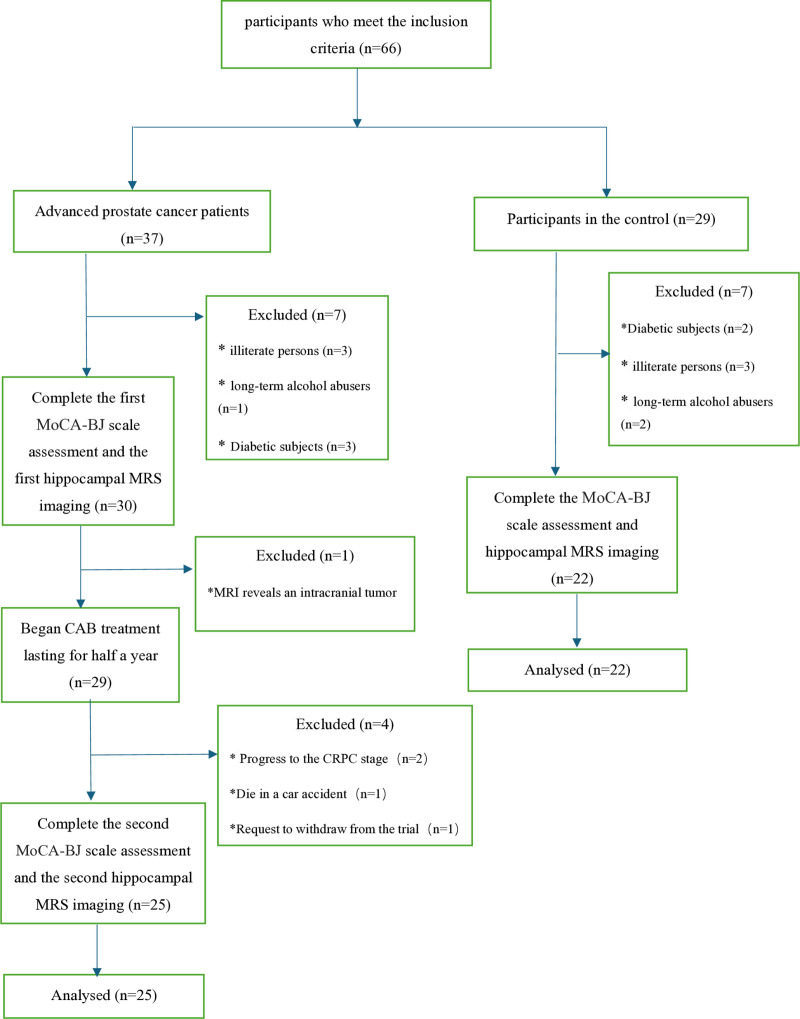
Flow diagram of the enrollment of participants in this study.

This study concluded on July 1st, 2024.The flow diagram of the enrollment of all participants in this study is detailed in Fig 1. All relevant data are within the manuscript and its Supporting information file(S1 File). The twenty-five patients with advanced PCa were included in CAB group for analysis, and 22 healthy cases were included in control group. The details of the participants’ information at the time of their visits are presented in Table 1. There were no significant differences in age, hemoglobin, education level, and initial blood testosterone levels between the two groups. The changes in relevant indicators (PSA, testosterone, and hs-CRP) in the CAB group after six months of CAB treatment are listed in S1 Table.

**Table 1:** Comparison of baseline data between the two groups.

Variables	CAB group(n = 25)	control group (n = 22)	*p*
Age(year), mean±s.d.	74.88 ± 6.72	74.59 ± 9.51	0.904
Gleason score^a^			
≤8	18		
9–10	7		
BMI(kg m^-2^), mean±s.d.	21.63 ± 1.44	21.94 ± 1.18	0.442
hypertension(yes/no)	6/19	6/16	0.797
HB (g/L), mean±s.d.	131.00 ± 10.73	131.09 ± 15.06	0.981
TNM stage			
≤T2C	6		
T3–T4	19		
Initial PSA(ng/ml)			
≤20	1	22	
20-100	17		
> 100	7		
Education			
≤ 6 y	19	18	0.897
> 6 y	6	4	
Initial hs-CRP(mg/l),Median(IQR)	15.00(11.07,44.00)	1.17(0.85,2.29)	＜0.001
Smoking(yes/no)	8/17	2/20	0.119
Initial tesosterone(nmol/l)	12.07 ± 2.40	12.46 ± 3.24	0.650

^a^The Gleason score is obtained by adding the grades of the two most common growth patterns observed in prostate cancer tissue. The total score ranges from 2 to 10. A higher Gleason score indicates a higher degree of aggressiveness and malignancy of the cancer cells.

BMI: body mass index; CAB group: the group of prostate cancer patients who underwent CAB treatment; HB: hemoglobin; hs-CRP: hypersensitive C-reactive protein; PSA: prostate specific antigen; s.d.: standard deviation.

The MoCA-BJ scores (including the total score and sub-item scores) of the two groups are recorded in Table 2. Before CAB therapy, there was no statistical difference in MoCA-BJ scores (including sub-item scores) between the participants in CAB group and control group. Compared with the control group, the CAB group exhibited significant decreases in attention (*P* = 0.04), delayed recall function (*P* = 0.02), and MoCA-BJ score (*P* = 0.03) after 6 months of CAB treatment. Six months after CAB treatment, the MoCA-BJ score(*P* = 0.00), delayed recall function(*P* = 0.00), visuospatial/executive function(*P* = 0.03) and attention function(*P* = 0.00) of patients in the CAB group, significantly decreased compared with those before CAB treatment.

**Table 2:** Comparison of MoCA-BJ scores among different groups(mean±s.d.).

	Pre-CAB	Post-CAB	Control group	*P* ^a^	*P* ^b^	*P* ^c^
V/E function	3.60 ± 0.50	3.32 ± 0.69	3.50 ± 0.59	0.53	0.34	0.03
Name	2.92 ± 0.27	2.88 ± 0.33	2.91 ± 0.29	0.89	0.75	0.57
Attention	3.64 ± 0.57	3.16 ± 0.62	3.77 ± 0.75	0.49	0.04	0.00
Language	2.08 ± 0.28	1.96 ± 0.45	2.18 ± 0.39	0.31	0.08	0.83
Abstraction	1.32 ± 0.47	1.20 ± 0.41	1.31 ± 0.48	0.99	0.36	0.18
Delayed recall	3.56 ± 0.71	2.88 ± 0.67	3.36 ± 0.79	0.37	0.02	0.00
Orientation	4.96 ± 0.45	4.76 ± 0.59	4.91 ± 0.61	0.74	0.40	0.13
Total score	22.08 ± 1.63	20.24 ± 1.42	21.95 ± 2.24	0.82	0.03	0.00

^a^Pre-CAB vs control group.

^b^Post-CAB VS control group.

^c^Pre-CAB VS Post-CAB.

V/E: Visuospatial/Executive.

**Fig 2:**
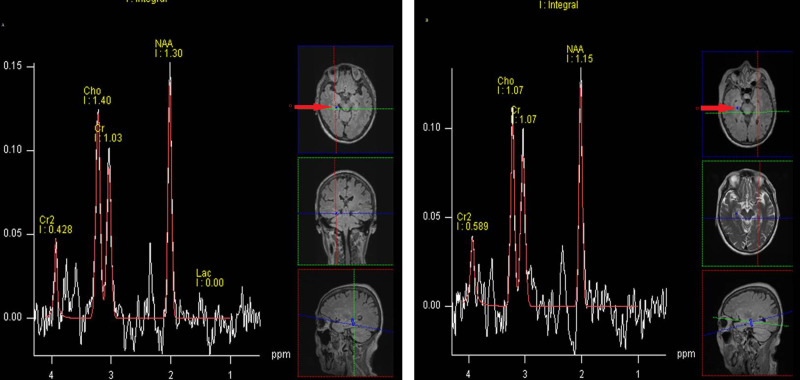
^1^H-MRS spectra of the right hippocampus in the same patient at different time points. A 65-year-old male patient with prostate cancer, TNM staging: T3bN1M1; Gleason score: 4+5. The patient has received continuous CAB therapy after being diagnosed with prostate cancer. **(A)**:MRS imaging of the right hippocampus before CAB therapy: NAA/Cr = 1.26, Cho/Cr = 1.36. **(B)**:MRS imaging of the right hippocampus after 6 months of CAB therapy: NAA/Cr = 1.07, Cho/Cr = 1.80; O: region of interest.

The bilateral hippocampal NAA/Cr and Cho/Cr of the two groups are recorded in Table 3. Before CAB therapy, there was no statistical difference in bilateral hippocampal NAA/Cr and Cho/Cr between the participants in CAB group and control group. Compared to the control group, the CAB group exhibited a significant increase in Cho/Cr in the right hippocampus after six months of CAB treatment. Six months after CAB treatment, the NAA/Cr ratios in both the left (*P* = 0.01) and right (*P* = 0.04) hippocampi of patients in the CAB group, significantly decreased compared with those before CAB treatment. Additionally, the Cho/Cr ratio in the left hippocampus significantly increased (*P* = 0.04). Example of ^1^H-MRS spectra is presented in Fig 2.

**Table 3:** Comparison of hippocampal MRS among different groups(mean±s.d.).

	Pre-CAB	Post-CAB	control group	*P* ^a^	*P* ^b^	*P* ^c^
NAA/Cr(Left)	1.10 ± 0.19	0.95 ± 0.23	1.09 ± 0.27	0.96	0.06	0.01
NAA/Cr(Right)	1.19 ± 0.24	1.11 ± 0.19	1.17 ± 0.29	0.71	0.38	0.04
Cho/Cr(Left)	1.12 ± 0.26	1.26 ± 0.29	1.19 ± 0.25	0.38	0.34	0.04
Cho/Cr(Right)	1.23 ± 0.42	1.31 ± 0.27	1.15 ± 0.22	0.42	0.03	0.31

^a^Pre-CAB vs control group.

^b^Post-CAB VS control group.

^c^Pre-CAB VS Post-CAB.

Spearman correlation analysis suggests that the MoCA-BJ score not only positively correlates with the NAA/Cr ratio in the left hippocampus but also positively correlates with initial blood testosterone levels. Further univariate linear regression analysis also confirms a positive correlation between the MoCA-BJ score and the NAA/Cr ratio in the left hippocampus (*β* = 5.4, *P* < 0.001), as well as a positive correlation with initial blood testosterone levels (*β* = 0.22, *P* = 0.026). However, in the multiple linear regression analysis, the MoCA-BJ score was only positively correlated with the NAA/Cr ratio in the left hippocampus (*β* = 4.66, *P* < 0.001), but there was no correlation with initial blood testosterone levels (*β* = 0.11, *P* = 0.194). The details are listed in S2 Table.

Furthermore, Spearman correlation analysis also indicates that there is a positive correlation between delayed recall function and both the NAA/Cr ratio in the left hippocampus and initial blood testosterone levels. Univariate linear regression analysis confirms a positive correlation between delayed recall function and the NAA/Cr ratio in the left hippocampus (*β* = 2.82, *P* < 0.001), as well as a positive correlation with initial blood testosterone levels (*β* = 0.09, *P* = 0.029). However, in the multiple linear regression analysis, the delayed recall function was only positively correlated with the NAA/Cr ratio in the left hippocampus (*β* = 2.76, *P* < 0.001), but there was no correlation with initial blood testosterone levels (*β* = 0.01, *P* = 0.721). The details are listed in S3 Table. In the aforementioned two multiple linear regression analyses, the collinearity analysis indicates that there is no collinearity relationship among all the mentioned independent variables.

The linear univariate analysis suggests a positive correlation between the initial blood testosterone level and the NAA/Cr ratio in the left hippocampus (*β* = 0.03, *P* = 0.017). Mediation Analyses indicate that testosterone influences MoCA-BJ total score (S1 Fig) and delayed recall function (S2 Fig) by affecting NAA/Cr in the left hippocampus.

## Discussion

There are currently few reports on whether PCa can lead to CI in patients, and the results are inconsistent [33–35]. Mohile et al found a high prevalence of lower than expected cognitive performance among a sample of patients just starting ADT for PCa [33]. However, other researchers found that the impact of PCa on cognitive performance could be negligible and the prevalence of CI was similar in patients with PCa and in the general population [34,35]. In this study, all the PCa patients included were at an advanced stage, and their baseline serum hs-CRP levels were significantly higher compared with the control group, confirming that these patients were in a chronic inflammatory state. However, no abnormalities were observed in the hippocampal biochemical metabolism and cognitive function of these patients when compared to the control group. Based on a comprehensive review of the literature, we believe that the following factors are relevant: first, systemic inflammatory status (such as elevated IL-6 and CRP) is not a necessary factor leading to cognitive impairment in patients; other conditions are also required, such as pre-existing brain pathology. For instance, mutations in the interleukin-6 receptor (IL-6R) within the brains of depression patients can lead to the activation of IL-6 trans-signaling pathway, subsequently triggering neuroinflammation and psychiatric disorders [23,36]. Animal studies have shown that when specific inhibitors targeting the trans-signaling pathway are used, disease-related behaviors caused by neuroinflammation are significantly reduced [37]. In the brains of patients with AD, pathological proteins such as amyloid-β (Aβ) and tau interact with circulating pro-inflammatory cytokines (like IL-6, TNF-α, and IL-1β), resulting in the activation and proliferation of microglia and astrocytes, which then continuously produce pro-inflammatory cytokines in the brain [38]. In our study, we had endeavored to exclude participants with potential pre-existing brain pathologies, such as AD, Parkinson’s disease, depression, chronic alcoholism, diabetes, etc. Therefore, despite the significantly elevated blood CRP levels observed in patients with advanced PCa compared with normal elderly males in this study, there were no notable differences in hippocampal biochemical metabolism and cognitive function when compared with the control group. Second, these PCa patients had normal androgen levels, which play a protective role for brain neurons [10]. In contrast, patients with AD and depression experienced a decline in their androgen concentrations, losing the neuroprotective effects of testosterone/dihydrotestosterone, which subsequently led to damage to brain neurons and a decline in cognitive function [10,14].

This study found that the left hippocampal NAA/Cr was positively correlated with delayed memory and total MoCA-BJ score, indicating that the normal number and function of neurons in the left hippocampus play a crucial role in maintaining memory and normal cognitive function in right-handed individuals. This is consistent with the report by Wang et al [39]. The reason why delayed memory function and total MoCA-BJ score are only correlated with the left hippocampus, we speculate, is that all the participants included in the study are right-handed, with a more developed left brain hemisphere [40,41]. This may result in a larger volume and richer blood supply in the left hippocampus compared to the right, which also explains why, in right-handed individuals, the left hippocampus is more susceptible to damage after the onset of functional brain diseases [42].

Currently, there are mixed opinions on whether endocrine therapy for PCa patients can lead to CI. Some studies have shown an increased risk, while others have found no association [35,43–45]. The reasons for the inconsistent results, as suggested by literature, can be speculated as follows. First, the patients included in these studies did not receive a unified endocrine therapy regimen. For example, some patients received ADT therapy alone, while others received CAB therapy. Second, in some studies, PCa patients who received intermittent endocrine therapy were included, and the detection of their cognitive function might be conducted during the treatment gap, when the cognitive function of patients has been improved with the recovery of circulating testosterone. Third, there may exist a selection bias among the enrolled PCa patients [35]. In this study, we found that after CAB therapy, advanced PCa patients exhibited cognitive decline, which was correlated with decreased NAA/Cr levels in both hippocampi and increased Cho/Cr levels in the left hippocampus, when compared with their pre-treatment levels. We speculate that the reasons for the decrease in NAA in both hippocampi of PCa patients are as follows. Firstly, the present study revealed that following CAB treatment, the serum testosterone levels in PCa patients experienced a substantial decline of approximately 300 times from their baseline values. This significant drop was anticipated to result in a decrease in blood supply to the bilateral hippocampus, a region known for its heightened sensitivity to ischemia [3]. Second, a sharp decrease in the concentration of testosterone in the circulation can also lead to a significant decline in the concentration of testosterone in the hippocampus, despite the fact that hippocampal neurons have the capability to synthesize testosterone themselves [46,47]. The self-synthesis of testosterone by hippocampal neurons is regulated by various factors such as substrate availability, enzyme activity, and hormonal feedback mechanisms [48]. In situations where the concentration of testosterone in the circulation drops sharply, these regulatory mechanisms may not be sufficient to maintain a stable level of testosterone within the hippocampus [46]. Third, the ARs located in the hippocampus were blocked by bicalutamide. Due to these combined factors, there was a reduction in the number of hippocampal neurons (apoptosis) and a decline in their function. Regarding the reason why only the Cho/Cr ratio in the left hippocampus increases, we speculate that the possible reasons are as follows. Firstly, all the patients included in this study are right-handed, which means they tend to have a larger left hippocampus with more distributed neurons [40,41]. Consequently, the damage caused by CAB treatment was more severe in the left hippocampus, leading to the apoptosis of more neurons and the breakdown of cell membranes. Secondly, neuronal apoptosis can stimulate glial cell proliferation [49]. Given the richer blood supply to the left hippocampus and the apoptosis of more neurons, the proliferation of glial cells may be more pronounced. Glial cell proliferation increases the synthesis and release of Cho, as Cho is an important precursor of cell membrane phospholipids, and glial cells are rich in cell membrane structures [50]. Therefore, the increase in the Cho/Cr ratio in the left hippocampus may be related to glial cell proliferation and accelerated cell membrane turnover.

After six months of CAB therapy, the MoCA-BJ total score (20.24 ± 1.42 *vs* 21.95 ± 2.24, *P* = 0.03) and delayed recall function (2.88 ± 0.67 *vs* 3.36 ± 0.79, *P* = 0.02) of patients with advanced PCa significantly declined compared with the control group. However, despite a notable decreasing trend observed in the left hippocampal NAA/Cr ratio of PCa patients after six months of CAB therapy compared to the control group, there was no statistically significant difference (0.95 ± 0.23 *vs* 1.09 ± 0.27, *P* = 0.06). Regarding the reason, we speculate that it may be related to the relatively small sample size.

In this study, Spearman correlation analysis suggestes that there is no correlation between the two cognitive domains of visuospatial/executive function and attention function and the NAA/Cr in bilateral hippocampi. However, after CAB treatment, the scores of visuospatial/executive function, as well as attention function, in the PCa group significantly decreased compared to those before CAB treatment. We believe that the mechanism leading to this change is still related to the sharp decline in androgen concentration and the blockage of ARs. As reported in the literature, ARs are not only distributed in the hippocampus but also abundantly present in the cerebral cortex [7]. For example, when testosterone binds to ARs located in the prefrontal cortex, it can induce the formation of dendritic spines and synapses in the prefrontal cortex [2].The literature suggests that attention is intimately tied to the functioning of the prefrontal cortex, and similarly, visuospatial/executive functions are closely associated with the functioning of the occipital cortex [2]. Therefore, we speculate that after CAB treatment, not only will the number of bilateral hippocampal neurons decrease and their activity decline in PCa patients [4–6,51], but also the biochemical metabolism of the prefrontal and occipital cortices may decrease, potentially leading to neuronal apoptosis in these areas, and further resulting in the decline of cognitive functions such as visuospatial/executive function, as well as attention [2,52,53].

One of several limitations of this study is the small sample size. Second, there was a lack of real control group in the PCa group, because it was ethically unacceptable. When advanced PCa is diagnosed, CAB therapy is the best treatment option. We cannot ask patients to wait for half a year before receiving CAB therapy just for the sake of this research. Third, despite the abundance of existing research confirming that there are no significant changes in cognitive function and brain structure (or function) within a six-month period among normal elderly males [52,54–58], it remains necessary to reassess the cognitive function and perform bilateral hippocampal MRS imaging for participants in the control group six months after the initial examination. However, due to funding constraints and the lack of cooperation from participants, it was not possible to establish a temporal comparison within the control group. Lastly, due to the limitations of our center, we were unable to detect IL-6, and therefore, there are no relevant data on IL-6 in this study.

## Conclusions

In summary, the impact of advanced PCa on cognitive performance could be negligible. However, patients experienced secondary bilateral hippocampal injury after CAB treatment, which further led to cognitive dysfunction.^1^H-MRS can detect early metabolic changes in the corresponding brain functional areas of these patients, providing a scientific basis for the diagnosis, prevention, and treatment of early cognitive dysfunction.

## Supporting information

S1 File Raw data of all participants in this study. (CSV)

S1 Table Changes in relevant indicators in CAB group after CAB treatment. hs-CRP: hypersensitive C-reactive protein; PSA: prostate specific antigen. (DOCX)

S2 Table Association between the left hippocampal NAA/Cr, testosterone, and the MoCA-BJ. CI, confidence interval. ^a^Association between the left hippocampal NAA/Cr and the MoCA-BJ(using the univariate linear regression). ^b^Association between the left hippocampal NAA/Cr and the MoCA-BJ(using the multiple linear regression; adjusted for age, education and tesosterone). ^c^Association between the initial blood testosterone levels and the MoCA-BJ(using the univariate linear regression). ^d^Association between the initial blood testosterone levels and the MoCA-BJ(using the multiple linear regression; adjusted for age, education and the left hippocampal NAA/Cr).
(DOCX)

S3 Table Association between the left hippocampal NAA/Cr, testosterone, and delayed recall function. CI, confidence interval. ^a^Association between the left hippocampal NAA/Cr and delayed recall function (using the univariate linear regression). ^b^Association between the left hippocampal NAA/Cr and delayed recall function (using the multiple linear regression; adjusted for age, education and tesosterone). ^c^Association between the initial blood testosterone levels and delayed recall function (using the univariate linear regression). ^d^Association between the initial blood testosterone levels and delayed recall function (using the multiple linear regression; adjusted for age, education and the left hippocampal NAA/Cr).
(DOCX)

S1 Fig Mediating effect of left hippocampal NAA/Cr between testosterone and MoCA-BJ (adjusted for age and education). Total effect: 0.2654 (0.0806, 0.4483), *P* = 0.004; Proportion of mediated: 0.5549 (0.183, 1.3917), *P* = 0.012. ACME, average causal mediation effects; ADE, average direct effects.
(TIF)

S2 Fig Mediating effect of left hippocampal NAA/Cr between testosterone and delayed recall function (adjusted for age and education). Total effect: 0.0977 (0.0206, 0.1737), *P* = 0.01; Proportion of mediated: 0.9039 (0.489, 1.7521), *P* = 0.014. ACME, average causal mediation effects; ADE, average direct effects.
(TIF)

S1 Checklist STROBE checklist. (DOCX)
