# Supplementary material for: 1H-MRS study of hippocampus in advanced prostate cancer patients: Relationship between hippocampal secondary damage and cognitive disorder following combined androgen blockade therapy
Source: PLoS One. 2025 May 7;20(5):e0323323. doi: 10.1371/journal.pone.0323323 (PMC12058151; doi:10.1371/journal.pone.0323323)
Supplement: S1 Table — (DOCX) [file pone.0323323.s002.docx]

| *v*ariables | Pre-CAB | Post-CAB | *p* |
| --- | --- | --- | --- |
| PSA(ng/ml),Median(IQR) | 51.20(38.50,120.18) | 0.07(0.02,0.26) | ＜0.001 |
| hs-CRP(mg/l),Median(IQR) | 15.00(11.07,44.00) | 1.40(0.94,2.05) | ＜0.001 |
| Tesosterone(nmol/l), mean±s.d. | 12.07±2.40 | 0.04±0.01 | ＜0.001 |
