# Supplementary material for: 1H-MRS study of hippocampus in advanced prostate cancer patients: Relationship between hippocampal secondary damage and cognitive disorder following combined androgen blockade therapy
Source: PLoS One. 2025 May 7;20(5):e0323323. doi: 10.1371/journal.pone.0323323 (PMC12058151; doi:10.1371/journal.pone.0323323)
Supplement: S2 Table — CI, confidence interval. aAssociation between the left hippocampal NAA/Cr and the MoCA-BJ(using the univariate linear regression). bAssociation between the left hippocampal NAA/Cr and the MoCA-BJ(using the multiple linear regression; adjusted for age, education and tesosterone). cAssociation between the initial blood testosterone levels and the MoCA-BJ(using the univariate linear regression). dAssociation between the initial blood testosterone levels and the MoCA-BJ(using the multiple linear regression; adjusted for age, education and the left hippocampal NAA/Cr). (DOCX) [file pone.0323323.s003.docx]

|  | NO. | Non-adjusted (*β*,95%CI) | *P* value | Adjusted (*β*,95%CI) | *P* value |
| --- | --- | --- | --- | --- | --- |
| NAA/Cr(Left) | 47 | 5.4 (3.55~7.25)^a^ | <0.001 | 4.66 (2.66~6.66)^b^ | <0.001 |
| Tesosterone | 47 | 0.22 (0.03~0.41)^c^ | 0.026 | 0.11 (-0.05~0.28)^d^ | 0.194 |
