# Supplementary material for: 1H-MRS study of hippocampus in advanced prostate cancer patients: Relationship between hippocampal secondary damage and cognitive disorder following combined androgen blockade therapy
Source: PLoS One. 2025 May 7;20(5):e0323323. doi: 10.1371/journal.pone.0323323 (PMC12058151; doi:10.1371/journal.pone.0323323)
Supplement: S3 Table — CI, confidence interval. aAssociation between the left hippocampal NAA/Cr and delayed recall function (using the univariate linear regression). bAssociation between the left hippocampal NAA/Cr and delayed recall function (using the multiple linear regression; adjusted for age, education and tesosterone). cAssociation between the initial blood testosterone levels and delayed recall function (using the univariate linear regression). dAssociation between the initial blood testosterone levels and delayed recall function (using the multiple linear regression; adjusted for age, education and the left hippocampal NAA/Cr). (DOCX) [file pone.0323323.s004.docx]

|  | NO. | Non-adjusted (*β*,95%CI) | *P* value | Adjusted (*β*,95%CI) | *P* value |
| --- | --- | --- | --- | --- | --- |
| NAA/Cr(Left) | 47 | 2.82 (2.35~3.29)^a^ | <0.001 | 2.76 (2.23~3.29)^b^ | <0.001 |
| Tesosterone | 47 | 0.09 (0.01~0.16)^c^ | 0.029 | 0.01 (-0.04~0.05)^d^ | 0.721 |
