# Supplementary figures and images for: 1H-MRS study of hippocampus in advanced prostate cancer patients: Relationship between hippocampal secondary damage and cognitive disorder following combined androgen blockade therapy
Source: PLoS One. 2025 May 7;20(5):e0323323. doi: 10.1371/journal.pone.0323323 (PMC12058151; doi:10.1371/journal.pone.0323323)

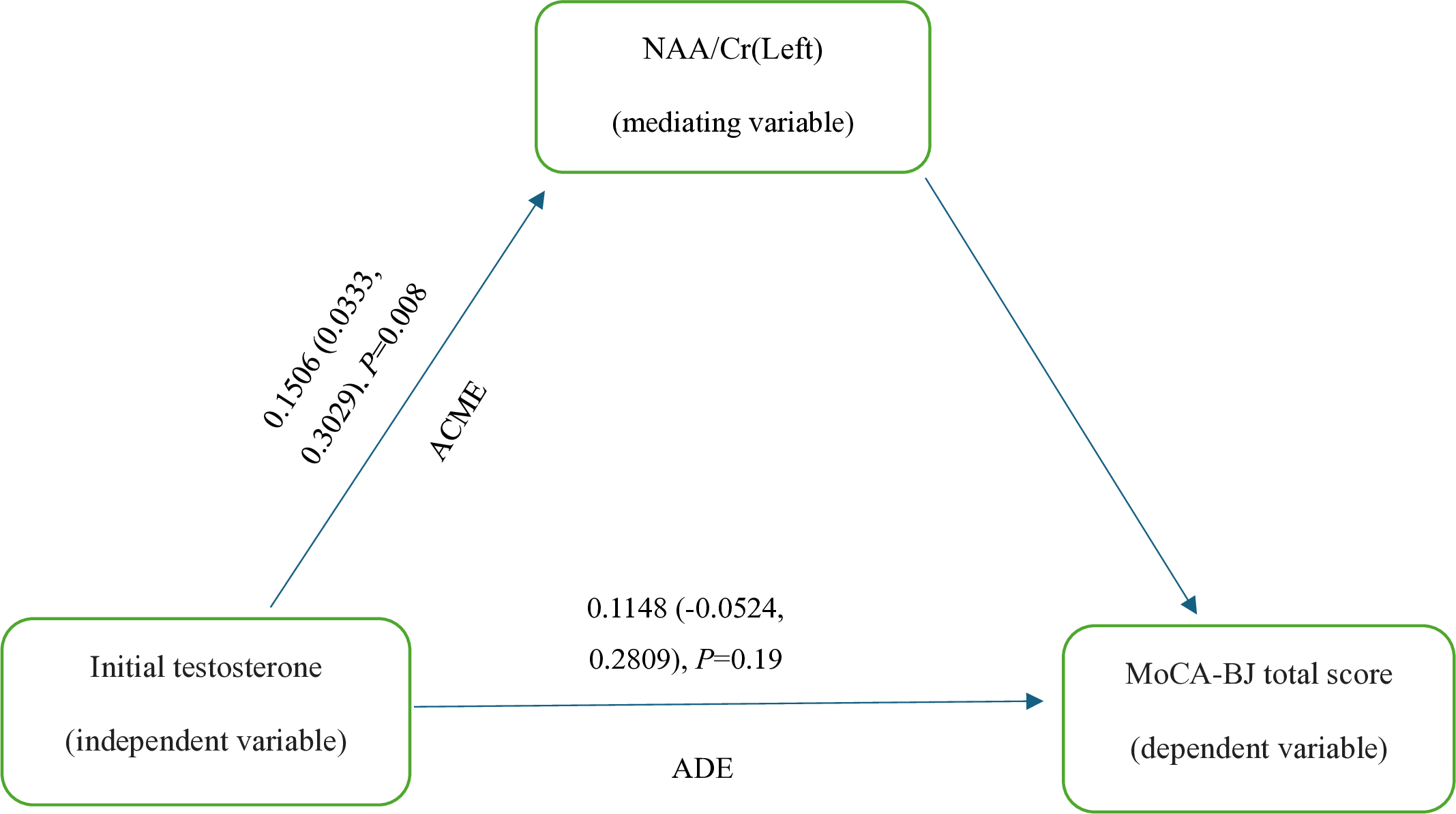

Supplement: S1 Fig — Total effect: 0.2654 (0.0806, 0.4483), P = 0.004; Proportion of mediated: 0.5549 (0.183, 1.3917), P = 0.012. ACME, average causal mediation effects; ADE, average direct effects. (TIF) [file pone.0323323.s005.tif]

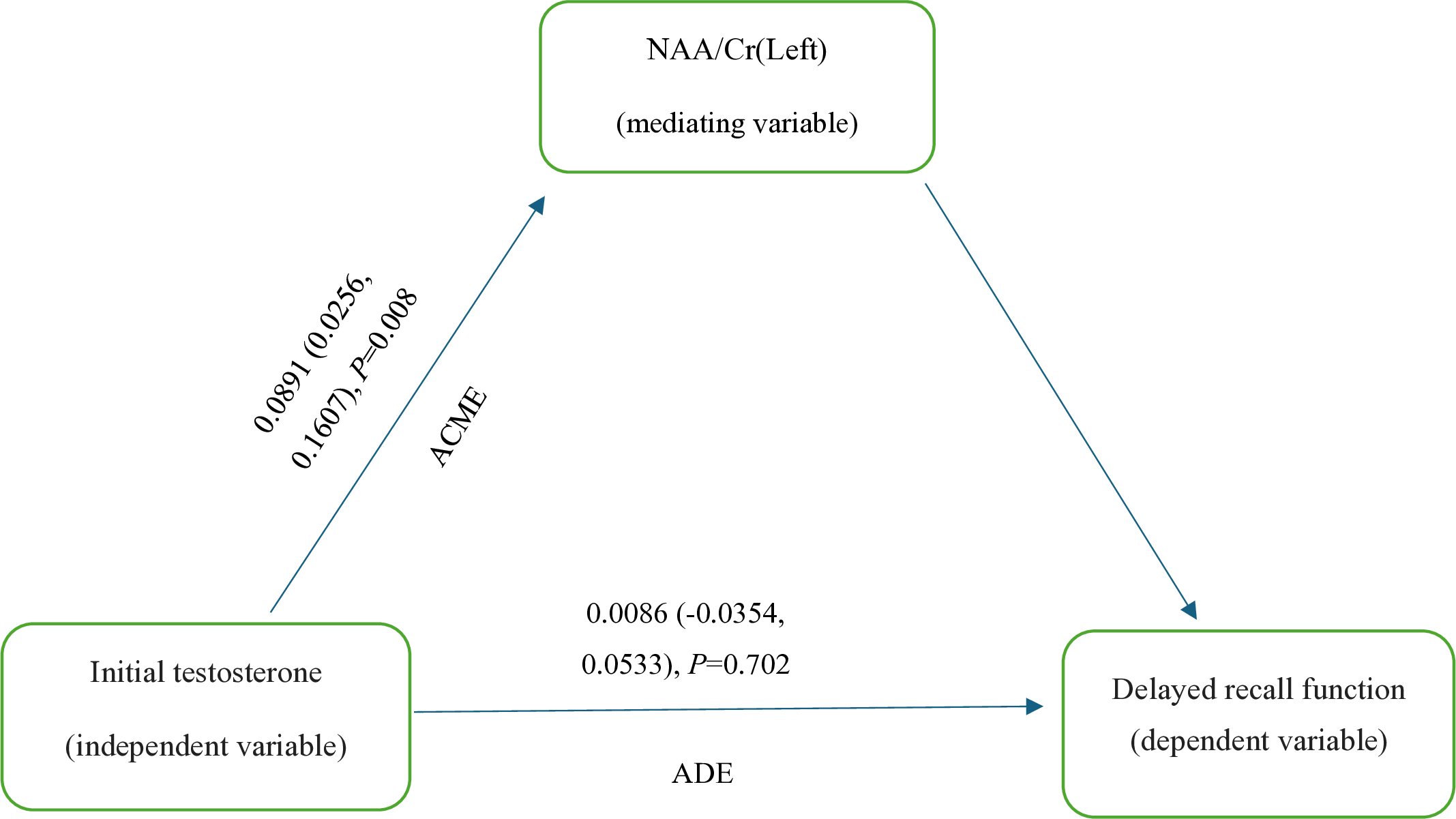

Supplement: S2 Fig — Total effect: 0.0977 (0.0206, 0.1737), P = 0.01; Proportion of mediated: 0.9039 (0.489, 1.7521), P = 0.014. ACME, average causal mediation effects; ADE, average direct effects. (TIF) [file pone.0323323.s006.tif]
